# Regulation of CD44v6 expression in gastric carcinoma by the IL-6/STAT3 signaling pathway and its clinical significance

**DOI:** 10.18632/oncotarget.17435

**Published:** 2017-04-26

**Authors:** Yuan-Yuan Xu, Ming Guo, Liu-Qing Yang, Fan Zhou, Cao Yu, Aixiu Wang, Tao-Hong Pang, Hong-Yan Wu, Xiao-Ping Zou, Wei-Jie Zhang, Lei Wang, Gui-Fang Xu, Qin Huang

**Affiliations:** ^1^ Department of Gastroenterology, Nanjing Drum Tower Hospital Affiliated to Nanjing University Medical School, Nanjing, Jiangsu, China; ^2^ Department of Gastroenterology, People's Hospital of Anji, Huzhou, China; ^3^ Department of Pathology, Nanjing Drum Tower Hospital Affiliated to Nanjing University Medical School, Nanjing, Jiangsu, China; ^4^ Department of Gastrointestinal Surgery, Nanjing Drum Tower Hospital Affiliated to Nanjing University Medical School, Nanjing, Jiangsu, China; ^5^ Department of General Surgery, Nanjing Drum Tower Hospital Affiliated to Nanjing Medical University, Nanjing, Jiangsu, China; ^6^ Department of Pathology, VA Boston Healthcare System and Harvard Medical School, Boston, MA, United States

**Keywords:** CD44v6, cancer stem cell, gastric carcinoma, IL-6, STAT3

## Abstract

As a cancer stem cell marker, CD44 variant 6 (CD44v6) has been implicated in carcinogenesis, tumor progression, and metastasis in a variety of human carcinomas. However, little is known about the expression of CD44v6 in Gastric Carcinoma (GC). Therefore we investigated CD44v6 expression in clinical specimen and further explore the underlying molecular mechanisms.

In this study, we systemically investigated CD44v6 expression by immunohistochemistry in normal, premalignant gastric mucosa (low and high grade intraepithelial neoplasia), and GC at various stages. The correlation of CD44v6 expression with clinicopathological characteristics, and prognosis in GC was also analyzed. Next, we investigated cell proliferation, migration and invasion in GC cell lines. Furthermore, we explored a novel mechanism by which CD44V6 was upregulated in GC cell.

The immunohistochemistry results showed that enhanced expression of CD44v6 was closely associated with tumor differentiation, lymph node metastasis, TNM stage and poor prognosis in GC patients. In gastric cancer cell lines, CD44v6 involved in cell proliferation, invasion and metastasis in Next, report on a novel mechanism by which interleukin-6/signal transducer and activator of transcription 3 (IL-6/STAT3) signaling up-regulates expression of CD44v6. RNA interference silencing of STAT3 resulted in decrease of CD44v6 levels. We also found that STAT3 inhibitor AG490 decrease expression of CD44v6 by blocking activation of STAT3, even in the presence of IL-6. Targeting STAT3-mediated CD44v6 up-regulation may represent a novel, effective treatment by eradicating the stomach tumor microenvironment.

## INTRODUCTION

Gastric carcinoma (GC) is the fourth most common malignancy and the third leading cause of cancer-related death world-wide [1, 2]. In spite of recent progress in the surgical treatment and chemotherapy, the prognosis of GC patients remains poor [3]. Tumor recurrence including metastasis is the main cause of cancer-related death. Unfortunately, tumor recurrence after radical gastrectomy with curative intent is relatively common, occurring in 20% to 50% of GC patients [4, 5]. At present, the underlying molecular mechanisms responsible for tumor recurrence have not been fully elucidated. The specific tumor markers in detection of tumor recurrence stay elusive. Over the past decades, several cancer stem cells (CSCs) have been identified and characterized in various types of human cancers [6–9]. Because CSCs possess the ability to initiate tumorigenesis, promote progression, and resist conventional chemotherapies [8, 10, 11], the concept that CSCs are responsible for tumor initiation is quite well established. However, the role of CSCs in tumor recurrence remains poorly understood, especially in GC [12, 13].

The cell adhesion molecule, CD44, is a trans-membrane glycoprotein that binds hyaluronic acid, facilitates tumor invasion, and promote tumor recurrence [14–16]. A growing body of evidence suggests that CD44 is also a major cell surface marker of CSCs in several solid tumors, including GC [9, 12, 15–18]. Previous molecular studies show that CD44 is expressed as a standard form (CD44s) and also in numerous variants (CD44v) generated by alternative mRNA splicing [14, 17, 19]. Among CD44 variants, CD44 variant 6 (CD44v6) has been implicated in carcinogenesis, tumor progression, and recurrence in a variety of human cancers [20–25], identified recently as the marker of CSCs in brain tumors, colorectal and bladder carcinomas [21, 26, 27]. Overexpression of CD44v6 found to be an indicator of poor prognosis in hepatocellular carcinoma [28] and GC [29].

Despite the immense clinical importance, molecular mechanisms underlying expression of CD44v6 in GC are unknown. In GC and several other human malignancies in the liver, breast, head and neck, and hematogenetic organs, aberrant activation of signal transducer and activator of transcription 3 (STAT3) has been discovered [30, 31]. STAT3 is critical in inflammation-associated tumorigenesis by regulating numerous oncogenic and inflammatory genes [32], such as interleukin-6 (IL-6), a potent STAT3 activator, and highly expressed in response to hepatitis viral infection and systemic inflammation in the liver [33]. The IL-6/STAT3-signaling pathway has been previously reported to be involved in hepatic inflammation/regeneration, but unknown in GC, in which inflammation is one of the most prominent clinicopathologic characteristics. In this study, we hypothesized that CD44v6 played an important role in GC proliferation, invasion, and migration, and CD44v6 was upregulated via the IL6/STAT3-medicated pathway.

## RESULTS

### The expression of CD44v6 and pSTAT3 in gastric benign mucosa, premalignant lesion, and carcinoma

**Figure 1:**
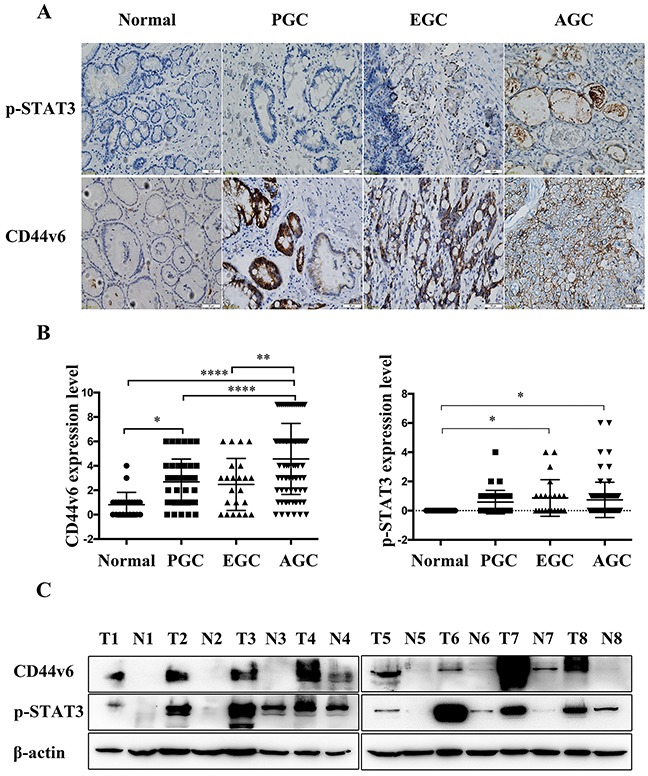
(**A**) Immunostaining and (**B**) analysis of the results of CD44v6, and pSTAT3 expression in Normal, precancerous lesions (PGC), early gastric cancer (EGC) and advanced gastric cancer (AGC) tissues (*P<0.05, **P<0.01, ****P<0.005); (**C**) Western blot analysis of CD44v6 and pSTAT3 expression in 8 paired advanced gastric carcinoma tumors (T1, T2, T3, T4, T5, T6, T7, T8) and corresponding adjacent normal gastric mucosal tissues (N1, N2, N3, N4, N5, N6, N7, N8).

By immunohistochemistry, CD44v6 is mainly membranous and p-STAT3 is mainly nuclear. The expression of CD44v6 was significantly progressively increased from minimal immunoreactivity in normal mucosa (0.82±1.006, mean± standard deviation) to moderate in premalignant lesion (2.68±1.887), early GC (2.48±2.129) and marked in advanced GC (4.56±2.912) (p<0.01). A similar immunoreactivity pattern was found with pSTAT3 immunostaining (0.00±0, 0.59±0.805, 0.87±1.254, 0.74±1.209, respectively)(p<0.05). Expression of CD44v6 and pSTAT3 were significantly elevated in different stages of GC, as shown in Figure 1A, B and Supplementary Table 1. The results of Western blotting in 8 paired advanced GC tumors and corresponding adjacent normal tissues confirmed significantly increased immunoreactivity of CD44v6 and pSTAT3 in advanced GC tissues, compared to normal tissue controls (Figure 1C).

### Correlation with clinicopathological characteristics and prognosis

**Figure 2:**
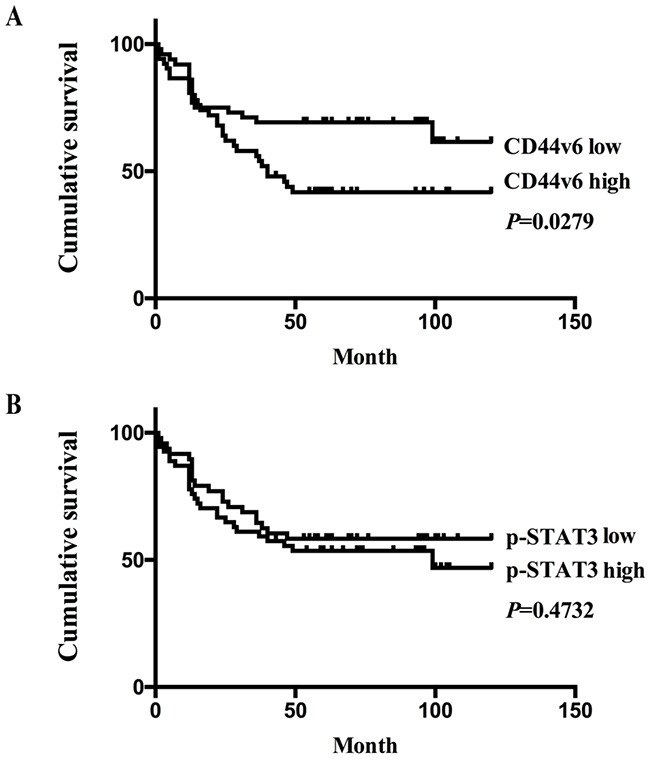
(**A**) The Kaplan-Meier survival analysis showed significantly poor prognosis in GC patients with high expression of CD44v6,compared to those with low-expression (P<0.05); (**B**) in contrast, there was no significant survival differences between GC patients with high or low expression of pSTAT3 (P>0.05), on the basis of immunohistochemistry results.

As shown in Table 1, CD44v6 expression in 103 GC tumors was associated with differentiation grade, depth of invasion, nodal metastasis, and TNM stage (p<0.005). However, there was no significant correlation between CD44v6 expression and other pathological parameters, such as patient age, gender, or tumor size. By the Kaplan–Meier method for post-resection survival analysis. GC patients with low expression of CD44v6 had a significantly better outcome than those with high expression of CD44v6 (P<0.05; Figure 2A). However, expression of pSTAT3 showed neither significant correlation with clinicopathology (Supplementary Table 2) nor prognosis (Figure 2B).

**Table 1:** The relationship between expression of CD44v6 and clinicopathological features in GC

Parameters	N	CD44v6		χ^2^-value	*P* value
		Low(%)	High(%)		
Gender					
Male	69	40(58.0)	29(42.0)	0.007	0.934
Female	34	20(58.8)	14(41.2)		
Age					
<60	48	25(52.1)	23(47.9)	1.407	0.236
≥60	55	35(63.6)	20(36.4)		
Size					
<6.0(cm)	80	50(62.5)	30(37.5)	2.658	0.103
≥6.0(cm)	23	10(43.5)	13(56.5)		
Differentiation					
Poor	49	21(42.9)	28(57.1)	9.109	0.003*
Well/moderate	54	39(72.2)	15(27.8)		
Depth of invasion					
T1/T2	41	32(78.0)	9(22.0)	10.976	0.001*
T3/T4	62	28(45.2)	34(54.8)		
Lymph node status					
No	42	32(76.2)	10(23.8)	9.383	0.002*
Yes	61	28(45.9)	33(54.1)		
TNM stage					
I/II	51	37(72.5)	14(27.5)	8.490	0.004*
III/IV	52	23(44.2)	29(55.8)		

### Proliferate roles of CD44v6 in gastric cancer cell line

By Western blotting, protein expression of CD44v6 in four GC cell lines was compared with one normal gastric epithelial cell line, GES-1. As exhibited in Supplementary Figure 1, CD44v6 protein expression was significantly higher in 3 GC cell lines, compared to that in control. However, CD44v6 expression was not significantly increased in the N87 GC cell line (Supplementary Figure 1). This inconsistency may be due to the different genetic background of cell lines (Supplementary Table 3).

**Figure 3:**
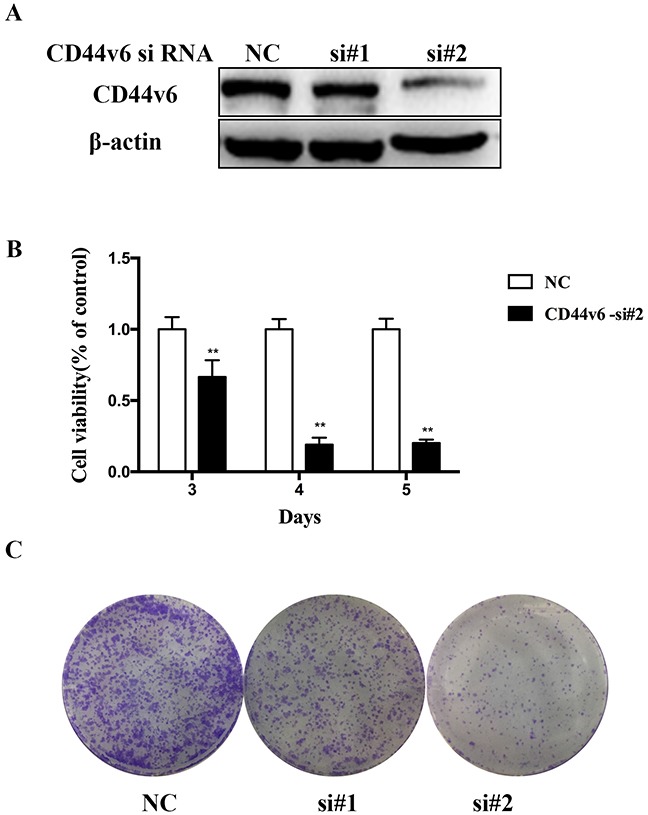
Investigation of the oncogenic functions of CD44v6 HGC-27 cells were transfected with negative-contronl siRNA and CD44v6-siRNA for 3 days. (**A**) The expression of CD44v6 and β-actin was detected by Western blot. (**B**) Proliferation of HGC-27 was examined using CCK8 assay. Cell viability was calculated by the following formula: relative cell viability = (absorbance450nm of treated group − absorbance450nm of blank)/(absorbance450nm of control group − absorbance450nm of blank). (**C**) Colony-forming ability of HGC-27 cell was investigated by colony formation assay (*P <0.05).

In order to investigate the functional roles of CD44v6 in GC, we first created two HGC-27 cell lines pre-transfected with CD44v6-siRNA (si#1,si#2) and negative control-siRNA (nc) (Figure 3A). Next, we compared the proliferation of these two cell lines using CCK-8 assay. The growth of cells of decreased CD44v6 is slower than negative-control cells, suggesting CD44v6 is capable of stimulating cell proliferation (Figure 3B). Similar results were observed in colony formation assays as CD44v6-downregulating cells displayed a lower colony-forming ability (Figure 3C).

### Effect of CD44v6 on expression of epithelial-mesenchymal transition molecules, gastric carcinoma cell migration and invasion *in vitro*

**Figure 4:**
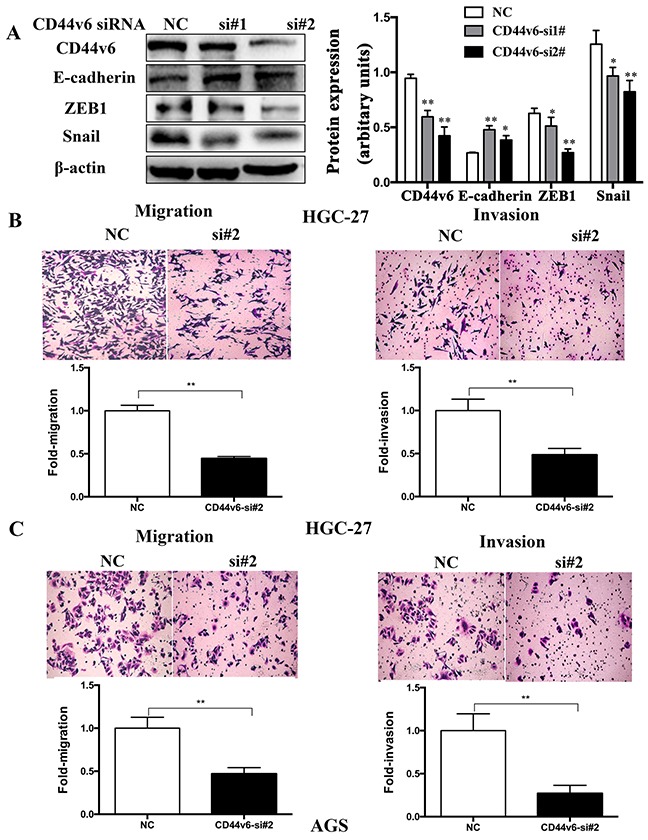
After knockdown of CD44v6 expression with siRNA (si#1,si#2) in the gastric cancer cell line, HGC-27, AGS (**A**) In HGC-27 cells, expression of epithelial-mesenchymal transition molecules, ZEB1 and Snail was markedly reduced, but E-cadherin expression increased. The right lane Further cell migration and invasion assays demonstrated conspicuous inhibition of (**B**) HGC-27 and (**C**) AGS cells after knockdown of CD44v6, compared to cells transfected with negative-si RNA (NC). The upper panel was the representative result images of cells. The lower panel was the quantitative comparison. NC was used as 1 for fold change calculation (*P <0.05, ** P <0.01).

In the GC cell line (HCG-27) after knockdown of CD44v6 with siRNA, decreased expression of CD44v6 was accompanied by downregulation of key epithelial-mesenchumal transition inducers, snail and zeb1, and by upregulation of E-cadherin (Figure 4A). Further investigation in HGC-27 (Figure 4B) and AGS (Figure 4C) cell lines demonstrated significant inhibition of the GC cell migration and invasion capability.

### CD44v6 expression by activation of the IL-6/STAT3 signaling pathway

**Figure 5:**
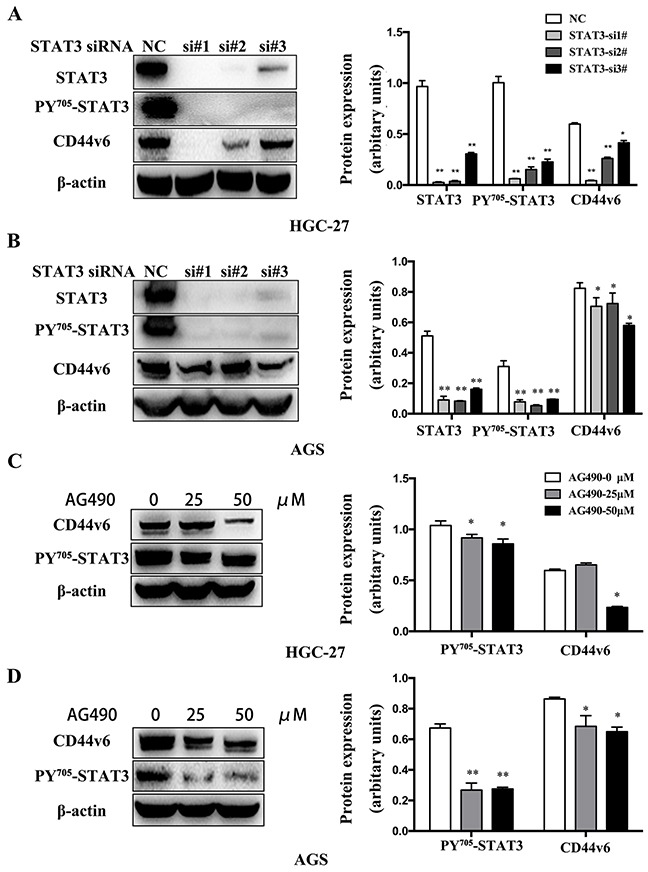
Western blot analysis of CD44v6 expression that was regulated by activation of STAT3 signaling (**A**) HGC-27 and (**B**) AGS cells were treated with STAT3 siRNA (si#1, si#2, si#3) for 3 days to knockdown STAT3 expression. As a result, CD44v6 expression was markedly reduced, compared to normal control cells (nc). In (**C**) HGC-27 and (**D**) AGS cells pretreated with AG490 to inactivate pan-JAK signaling to disable STAT3 for 12 hours, expression of CD44v6 was decreased (*P<0.05, ** P <0.01).

GC is known to be associated with chronic inflammation, in which IL-6-activated STAT3 expression is an important signaling pathway. To investigate whether pSTAT3 involves in activation of CD44v6 expression, we used the GC cell lines, HGC-27 and AGS with endogenously overexpressed CD44v6, to test if siRNA-mediated knockdown of STAT3 affected CD44v6 expression. As shown in Figure 4, a significantly decreased expression of CD44v6 after STAT3 knockdown was indeed observed (Figure 5A, 5B). Furthermore, functional inactivation of STAT3 by pan-JAK inhibitor AG-490 decreased the expression of CD44v6 (Figure 5C, 5D).

**Figure 6:**
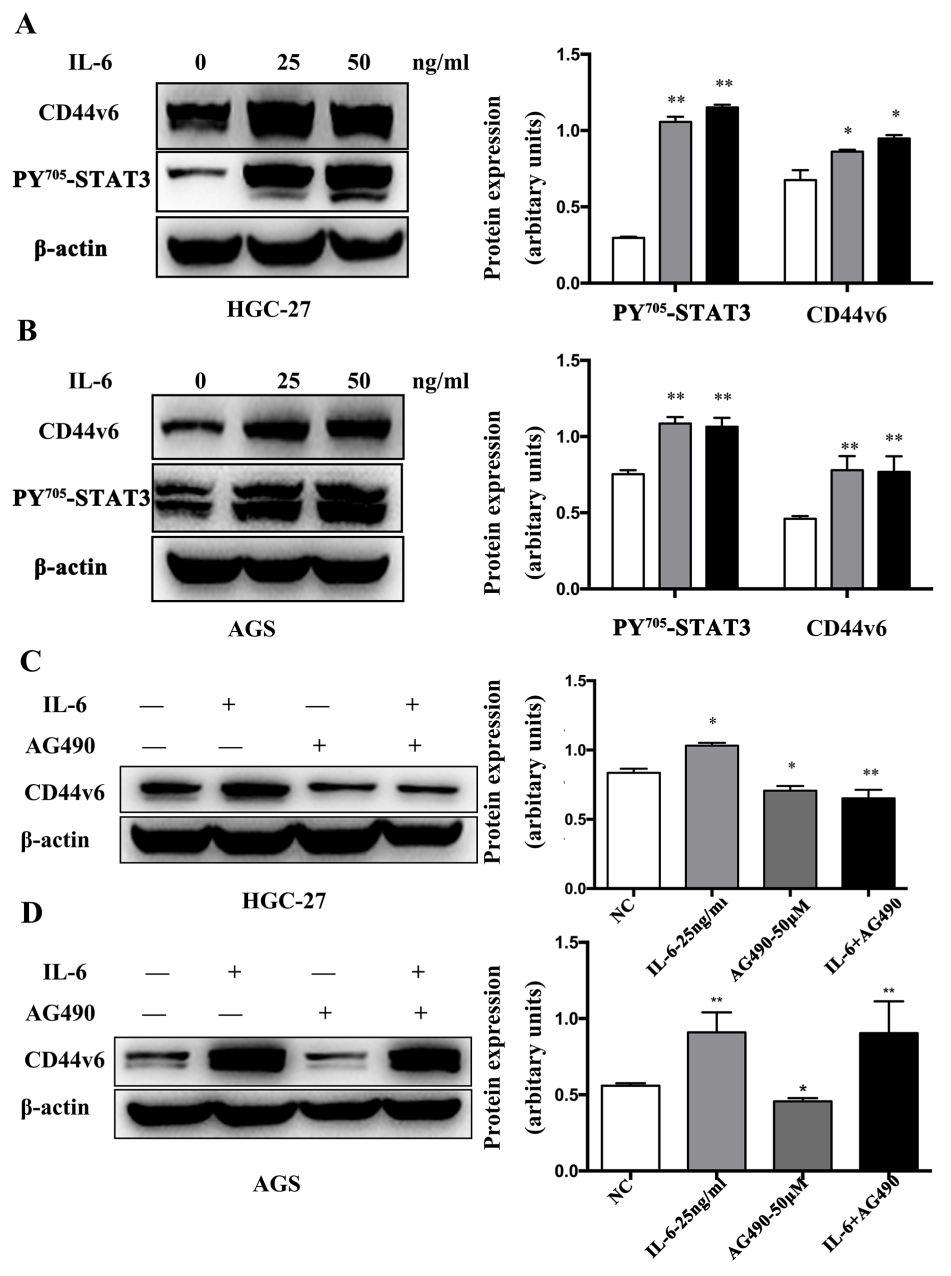
Western blot analysis of IL-6 induced CD44v6 expression through activation of STAT3 signaling (**A**) HGC-27 and (**B**) AGS cells were cultured in serum-free media for 6 hours. Then, cells were incubated with IL-6 for 6 hours, and expression of CD44v6 and p-STAT3 was dose-dependently increased. Pretreatment with AG490 (50μM) for 12 hours in (**C**) HGC-27 and (**D**) AGS cells to inactivate STAT3, next cultured in serum-free media for 6 hours and then incubation with IL-6 (50ng/ml) in a serum-free condition for 6 hours markedly reduced expression of CD44v6 (*P <0.05, ** P <0.01).

Next, we tested whether IL-6 treatment could modulate CD44v6 expression. As shown in Figure 6, IL-6 treatment resulted in not only high expression of CD44v6 but also pSTAT3 in both HGC-27 (Figure 6A) and AGS (Figure 6B) cells. The activation of STAT3 expression by IL-6 stimulation was also confirmed by Western blot in both HGC-27 (Figure 6A) and AGS cells (Figure 6B). Furthermore, a combination treatment with AG490 and IL-6 significantly reduced CD44v6 expression in HGC-27 cells (Figure 6C) but not in AGS cells (Figure 6D), suggesting that pSTAT3 is necessary for IL-6-induced CD44v6 expression in some gastric cancer cells. The difference of AGS and HGC-27 cells may be due to the different sensitivity to AG490 and IL-6. These results strongly imply that proinflammatory signaling can facilitate GC tumorigenesis through the IL-6/STAT3-mediated CD44v6 up-regulation.

## DISCUSSION

It is well established that the tumor CSC surface marker, CD44v6, is causally involved in cancer metastasis [29], correlated with tumorigenesis in some cancer types. For example, Mikami *et al*. reported that CD44v6 was overexpressed in extrahepatic bile duct carcinomas and linked to carcinoma differentiation [34]. In GC, Xin *et al*. first discovered that CD44v6-high expression was associated with poor survival in patients with advanced GC [35]. In 2014, two meta-analysis studies confirmed the association between high CD44v6 expression and worse overall post-resection survival in GC patients [36]. However, most studies have concentrated on advanced carcinomas, including GC. Studies focused on different stages of GC and functions of CSC marker, including CD44v6 and the molecular signaling pathways in tumor microenvironment are lacking.

In the present study, we studied the expression of CD44v6 and pSTAT3 in normal tissues, precancerous lesions, the early and advanced GC and discovered the progressive involvement of CD44v6 in GC tumorigenesis. The differences in CD44v6 expression between normal tissues, precancerous lesions, and advanced GC are statistically significant. In contrast, expression of pSTAT3 is significantly increased only in early and advanced GC tissue, compared to normal controls, as confirmed with both immunohistochemistry and Western blotting. The CD44v6 expression in normal mucosa was very low, but in premalignant lesions (including low and high grade intraepithelial neoplasia), the expression of CD44v6 was increased. Probably due to the premalignant lesions had many similar phenotype with the early GC, the CD44v6 expression was not significantly different between the two groups. However, the CD44v6 expression in advanced GC sharply elevated. This also showed the CD44v6 played a great role on the tumor progression. Importantly, CD44v6 expression is significantly predictive for poor prognosis in GC patients and correlated with poor tumor differentiation, deeper invasion, nodal metastasis, and advanced TNM stage. The findings of this study are compatible with the property of CD44v6 being an important prognosis marker in GC [29].

Despite the significant relevance of enhanced expression of CD44v6 to GC progression, little is known about the mechanisms of how it exerts its oncogenic function in GC. Herein, we investigated the functional roles of CD44v6 in GC cell proliferation, colony-forming ability, migration and invasion. The results are consistent with other two studies [29, 37], except for that CD44v6 did not activate cell migration in the scratch wound-healing assays [29]. The reason for this discrepancy maybe differences in background of GC cell lines and further studies will be needed to elucidate this question. That how CD44v6 involve in the cell proliferation, invasion and migration in GC also need further research.

Despite the studies on the CD44v6 expression, fundamental mechanisms underlying deregulating of CD44v6 are not known. The improved understanding of molecular pathway by which CD44v6 is deregulated in GC will provide useful information for elucidation of stomach CSC origin and development of novel treatment strategies against the deadly disease. IL-6 and its downstream molecules, such as STAT3, play an essential role in inflammation, aberrant immunity, and also carcinogenesis in some carcinomas [38–40]. IL-6 has been shown to enhance invasion of GC cells through sustained activation of STAT3 [31, 41, 42]. IL-6/STAT3-mediated CSC marker, CD133 up-regulation contributes to promotion of liver carcinoma [43]. Herein, we provide additional critical evidence on how IL-6/STAT3 signaling promotes GC invasion by inducing CD44v6 expression. In this study, a significantly decreased expression of CD44v6 after STAT3 knockdown was observed and functional inactivation of STAT3 by the pan-JAK inhibitor, AG-490, decreased the expression level of CD44v6 in both HGC-27 and AGS GC cells *in vitro*. The results suggest that IL-6 is able to activate expression CD44v6 and STAT3. On the other hand, obviously decreased CD44v6 expression in AG490-treated GC cells *in vitro* even with the concurrent IL-6 treatment suggests that activation of STAT3 is necessary for IL-6-induced CD44v6 expression. Therefore, our study results demonstrate that IL-6 is critical for induction of CD44v6 expression by STAT3 activation. IL-6 is also elevated in lots of cancers and is a potential regulator of stem cell renewal and proliferation [44–46]. However, the mechanism by which IL-6 regulates CD44v6 expression through STAT3 requires more detailed studies.

Taken together, our study results showed that CD44v6 is an important regulator of GC tumorigenesis, angiogenesis, and survival in an IL-6 mediated, pSTAT3-dependent manner; pSTAT3-mediated CD44v6 up-regulation may represent a promising target molecular signaling pathway for systemic therapy of human GC.

## MATERIALS AND METHODS

### Patients and tissue samples

One hundrend sixty six patients treated at the Nanjing Drum Tower Hospital in the Jiangsu Province, China, were enrolled over the period from Jan 2006 to Dec 2013, including 80 with GC, 23 with early GC staged at pT1, 41 with premalignant lesions (low and high grade intraepithelial neoplasia) in the gastric mucosa, and 22 normal controls. Patients without enough tissue sample or necessary clinicopathological information, or loss to follow-up were excluded from the study. The paired formalin-fixed paraffin-embedded tissue blocks were retrieved and recut for immunohistochemistry. Proteins were extracted with the conventional methods in fresh frozen matched tumor and non-tumor tissues stored in the Biobank at this hospital. The study protocol was approved by the Medical Ethics Committee of the Nanjing Drum Tower Hospital.

### Immunohistochemistry

Immunohistochemical (IHC) analysis for CD44v6, and p-STAT3 expression was performed on formalin-fixed, paraffin-embedded sections of surgical specimens. Briefly, sections were deparaffinized in xylene and rehydrated in gradient ethanol solutions up to distilled water. Endogenous peroxidase activity was blocked by 0.3% H_2_O_2_ in methanol for 20 min. The slides were immersed in 10mM citric buffer (pH 6.0) with heating for 15 min for antigen retrieval. Nonspecific binding sites were blocked with 10% normal goat serum for 10 min. Then, sections were incubated in a humidified chamber overnight with CD44v6 and p-STAT3 antibody. Immunostaining was visualized with Diaminobenzidine (DAB) and hematoxylin counterstain. The scoring for CD44v6 and p-STAT3 (expressed at a high level) was based on the area intensity score method (AIS) as previously descibed [49]. The protein expression was scored independently according to the intensity of cellular staining and the proportion of stained tumor cells. The staining intensity was scored as 0 (no staining), 1 (weak staining, light brown), 2 (moderate staining, yellow brown) and 3 (strong staining, brown). The proportions of stained tumor cells were graded as 0 (≤5 % positive cells), 1 (6–25 % positive cells), 2 (26–50 % positive cells) and 3 (≥51 % positive cells). The total scores for intensity and proportion were used to represent the level of protein expression. Positive controls consisted of each staining run and consisted of GCs known to express each of the antigens. Negative controls were normal mouse serum instead of the primary antibody.

### Reagents, siRNAs, and antibodies

Anti-CD44v6 (clone: ab78960) and anti-Snail (clone: ab82846) antibodies were purchased from Abcam (Cambridge, UK). Anti-phospho-(Tyr705)-STAT3 (p-STAT3) (clone: 4113; clone: 9131), E-cadherin(clone: 3195s) and ZEB1(clone:3396) antibodies were purchased from Cell Signaling Technology (Beverly, MA, USA). Anti-β-actin (clone: A5441) antibody was from Sigma-Aldrich (St Luis, MO, USA). AG490 were purchased from Selleck Chemicals (Houston, TX, USA). SiRNAs target STAT3 was purchased from Invitrogen (Carlsbad, CA, USA), and CD44v6 as well as a negative control siRNA (sequences are detailed in Supplementary Table 4) were purchased from (RiboBio, GuangZhou, China).

### Cell culture and transfection

The human gastric cancer cell lines, AGS and HGC-27, were purchased from the Cell Bank of Chinese Academy of Sciences, and were authenticated by China Center for Type Culture Collection (CCTCC) (Shanghai, China). All cell lines were cultured in Roswell Park Memorial Institute (RPMI)-1640 medium supplemented with 10% fetal calf serum (FBS) in a humidified incubator at 37°C with 5% CO2. Transfection was carried out using Lipofectamine RNAiMax Reagent (Invitrogen, California, USA) as described elsewhere (reverse transfection method) [47]. In brief, 50 pmol siRNA and 0.5 ml Opti-MEM I Medium (Invitrogen) without serum was mixed in each well of the six-well plate. Then 7.5 μl of Lipofectamine RNAiMAX reagent was added and gently mixed. After incubation for 20min at room temperature, 2ml of cell suspension including 3×10^5^ cells in complete growth medium without antibiotics was added into each well. This generated a final siRNA concentration of 20 nM.

### Western blot analysis

The total protein were extracted from target tissues and cells prepared with ice-cold lysis buffer (Biosharp). The supernatant was used for Western blot analysis. Protein concentrations were determined using a BCA protein kit (Beyotime Institute of Biotechnology, China). Samples containing equal amounts of protein were mixed with loading buffer containing 5% 2-mercaptoethanol and then heated for 10 min at 95°C. Twenty to forty micrograms of protein lysates were separated on 8–12 % sodium dodecyl sulfate-polyacrylamide gels and then transferred to the NC (Nitrocellulose) membranes (Millipore, Bedford, MA, USA). Tris Buffered Saline, with Tween-20 (TBST) containing with 5% nonfat milk or bovine serum albumin was used to block nonspecific binding for 2h at room temperature. Then, the membranes were incubated with the primary antibodies. Subsequently, the membranes were rinsed three times with Tris Buffered Saline (TBS) and 0.1% Tween-20 (TBS-T) for 10min and re-incubated for 1h at room temperature in blocking buffer with each Horseradish Peroxidase (HRP)-conjugated secondary antibody (1:5000), then washed three times for 10min each. Signals generated by enhanced chemiluminescence (Millipore) were recorded with a CCD camera (CLINX, Shanghai, China). Data are representative of at least three independent experiments.

### Cell proliferation

Cell viability was detected by Cell Counting Kit (CCK-8) assay. Cells pretreated with siRNA for 2 days were seeded into 96-well plates at 5 × 10^3^ cells/well and then cultured for 24h, 48h, and 72h at 37°C. After treatment for indicated times, 10 μl CCK-8 solutions were added to each well of the plate. Plates were incubated at 37°C for 1 h, and then the absorbance at 450 nm was measured. All experiments were carried out in triplicate and repeated three times independently.

### Colony formation assays

Colony-formation assay was performed as previously described [48]. In brief, the cell lines were transfected with CD44v6 siRNA (si#1,si#2), negative control-siRNA, for 3 days. Then 1 × 10^3^ cells/well were seeded into six-well plates. After 14 days, the colonies were fixed with methanol and stained with crystal violet. Crystal violet stained colonies were photographed. All experiments were done in triplicate and repeated three times independently.

### Cell migration and invasion analysis

Transfilter migration and invasion assays were performed on the HGC-27 and AGS cell lines in serum-free RPMI with 8.0-μm pore inserts on a 24-well Transwell (Corning Costar, Lowell, MA). The HGC-27 cell lines were transfected with CD44v6 siRNA, scRNA, for 3 days and then transferred to the upper chamber of the Transwell coated with 0.5mg/ml collagen type I and Matrigel (BD Bioscience, San Jose, CA) at 1:8 dilution. Migrating and invading cells were quantified after hematoxylin and eosin staining. Migration and invasion assays were performed after transfection, as previously described [50, 51].

### Statistical analysis

All statistical analyses were performed by the software “Statistical Package for Social Science” (SPSS) version 22.0 for Windows (SPSS, Chicago, IL, USA) and GraphPad Prism software v.6.01. Differences in expression of CD44v6, and pSTAT3 among advanced and early GC, gastric premalignant lesions, and normal gastric mucosal tissues were compared by the one-way analysis of variance (ANOVA) test. The associations among expression of CD44v6, pSTAT3, and clinicopathological characteristics were analyzed using the Students’ *t* test or the Chi-square test, where appropriate. The probability of survival was estimated by the Kaplan-Meier method with a log-rank test. All *P* values were two-sided and considered statistically significant if less than 0.05.

## SUPPLEMENTARY MATERIALS FIGURES AND TABLE


