# Supplementary material for: Regulation of CD44v6 expression in gastric carcinoma by the IL-6/STAT3 signaling pathway and its clinical significance
Source: Oncotarget. 2017 Apr 26;8(28):45848–61. doi: 10.18632/oncotarget.17435 (PMC5542232; doi:10.18632/oncotarget.17435)
Supplement: Supplementary file 1 [file oncotarget-08-45848-s001.pdf]

Regulation of CD44v6 expression in gastric carcinoma by the IL-6/STAT3 signaling pathway and its clinical significance

SUPPLEMENTARY MATERIALS

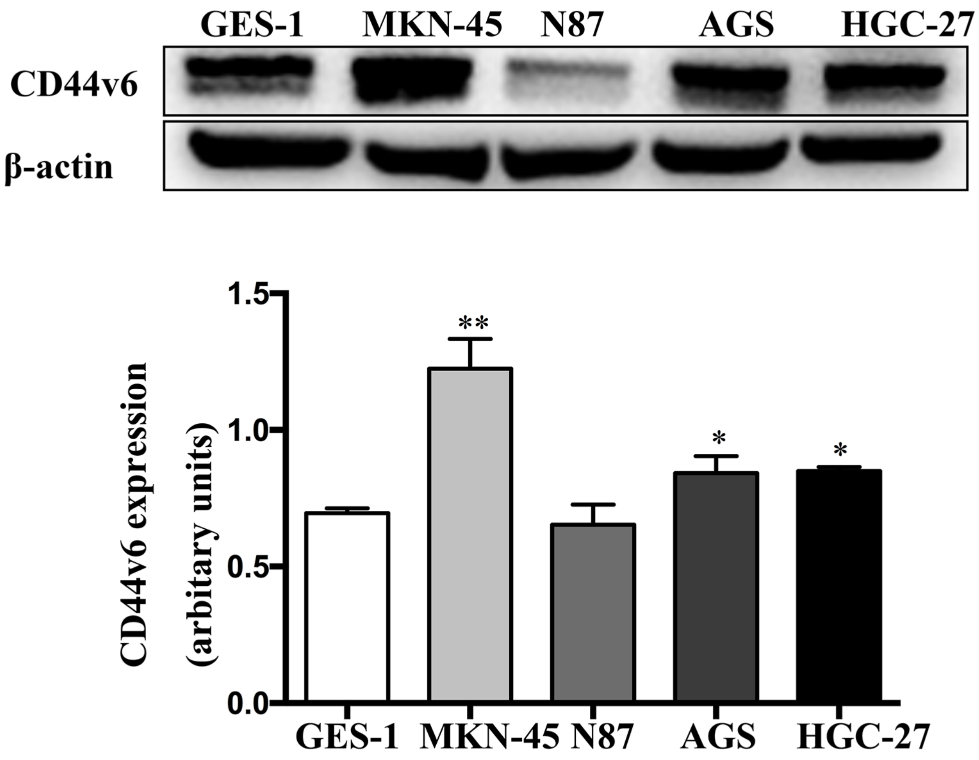

Supplementary Figure 1: Western blotting showed higher expression of CD44v6 in gastric cancer cell lines (MKN45, AGS, and HGC-27), except for N87, compared to that of normal gastric epithelial cell line GES-1 (*P <0.05, ** P <0.01).

Supplenmentary Table 1: The primer sequences of pSTAT3 and CD44v6 small interference RNA

	CD44v6		p-STAT3	
	$\bar{x} \pm s$	p	$\bar{x} \pm s$	p
Chronic gastistis	0.82±1.006	0.000[#]	0.00±0.000	0.000[#]
Precancerous leison	2.68±1.877		0.59±0.805	
Early gastric cancer	2.48±2.129		0.87±1.254	
Advanced cancer	4.56±2.912		0.74±1.209	

Supplenmentary Table 2: CD44v6 and p-STAT3 expression level were compared by ANOVA method

Parameters	N	p-STAT3		χ^2-value	*P* value
		Low(%)	High(%)		
Gender					
Male	69	34(49.3)	35(50.7)	1.428	0.232
Female	34	21(61.8)	13(38.2)		
Age					
<60	48	26(54.2)	22(45.8)	0.021	0.884
≥60	55	29(52.7)	26(47.3)		
Size					
<6.0(cm)	80	46(57.5)	34(42.5)	2.422	0.120
≥6.0(cm)	23	9(39.1)	14(60.9)		
Differentiation					
Poor	49	26(53.1)	23(46.9)	0.004	0.948
Well/moderate	54	29(53.7)	25(46.3)		
T satge					
T1/T2	42	25(59.5)	17(40.5)	1.069	0.301
T3/T4	61	30(49.2)	31(50.8)		
Lymphnode metastisis					
No	42	27(64.3)	15(35.7)	3.378	0.066
Yes	61	28(45.9)	33(54.1)		
TNM stage					
I/II	51	31(60.8)	20(39.2)	2.215	0.137
III/IV	52	24(46.2)	28(53.8)		

Supplenmentary Table 3: Background of different gastric cancer cell lines

Cell name	MKN-45	N87	AGS	HGC-27
Tissue and cell type	Diffuse adenocarcinoma_	–	Adenocarcinoma	Undifferentiated adenocarcinoma_
Morphology	Round	Epithelium	Epithelium	Epithelium
Growth characteristics	Adherent growth	Adherent growth	Adherent growth	Adherent growth
Gender	Female	Male	Female	-
Others	From stomach lymph nodes of a young woman with signet ring cell gastric cancer	–	Derived from an untreated resection of tumor fragments	Secrete mucin

The above information is derived from the following two web-sites:
(1) Chinese Academy of Sciences typical culture collection committee cell bank. http://www.cellbank.org.cn/index.asp
(2) Cancer Cell Line Encyclopedia.
https://portals.broadinstitute.org/ccle/home

Supplenmentary Table 4: The relationship between expression of p-STAT3 and clinicopathology of gastric carcinoma

Gene	Sequence
STAT3	
si#1	5'-GCCAATTGTGATGCTTCCCTGATTG-3'
si#2	5'-TGGCCCAATGGAATCAGCTACAGCA-3'
si#3	5'-GATAACGTCATTAGCAGAATCTCAA-3'
CD44v6	
si#1	5'- GCAACTCCTAGTAGTACAAdTdT-3'
si#2	5'- TGAGGGATATCGCCAAACAdTdT-3'
